# Association between clinical antibiotic resistance and susceptibility of *Pseudomonas* in the cystic fibrosis lung

**DOI:** 10.1093/emph/eow016

**Published:** 2016-05-21

**Authors:** Gunther Jansen, Niels Mahrt, Leif Tueffers, Camilo Barbosa, Malte Harjes, Gernot Adolph, Anette Friedrichs, Annegret Krenz-Weinreich, Philip Rosenstiel, Hinrich Schulenburg

**Affiliations:** ^1^ Evolutionary Ecology and Genetics, Zoological Institute, University of Kiel, Am Botanischen Garten 1-9, 24118 Kiel, Germany; ^2^ Fachklinik Satteldüne, Tanenwai 32, Nebel, 25946 Amrum, Germany;; ^3^ Department of Internal Medicine; ^4^ Department of Pharmacy, University Hospital Schleswig-Holstein, Kiel, Germany; ^5^ LADR GmbH Medizinisches Versorgungszentrum Plön, Krögen 6, 24306 Plön, Germany; ^6^ Institute for Clinical Molecular Biology, University of Kiel, Kiel, Germany

**Keywords:** cystic fibrosis, antibiotics, collateral sensitivity, cross-resistance, multi-drug resistance, clinical sampling depth, *Pseudomonas aeruginosa*

## Abstract

**Background and objectives:** Cystic fibrosis patients suffer from chronic lung infections that require long-term antibiotic therapy. *Pseudomonas* readily evolve resistance, rendering antibiotics ineffective. *In vitro* experiments suggest that resistant bacteria may be treated by exploiting their collateral sensitivity to other antibiotics. Here, we investigate correlations of sensitivity and resistance profiles of *Pseudomonas aeruginosa* that naturally adapted to antibiotics in the cystic fibrosis lung.

**Methodology:** Resistance profiles for 13 antibiotics were obtained using broth dilution, E-test and VITEK mass spectroscopy. Genetic variants were determined from whole-genome sequences and interrelationships among isolates were analyzed using 13 MLST loci.

**Result:** Our study focused on 45 isolates from 13 patients under documented treatment with antibiotics. Forty percent of these were clinically resistant and 15% multi-drug resistant. Colistin resistance was found once, despite continuous colistin treatment and even though colistin resistance can readily evolve experimentally in the laboratory. Patients typically harbored multiple genetically and phenotypically distinct clones. However, genetically similar clones often had dissimilar resistance profiles. Isolates showed mutations in genes encoding cell wall synthesis, alginate production, efflux pumps and antibiotic modifying enzymes. Cross-resistance was commonly observed within antibiotic classes and between aminoglycosides and β-lactam antibiotics. No evidence was found for consistent phenotypic resistance to one antibiotic and sensitivity to another within one genotype.

**Conclusions and implications:** Evidence supporting potential collateral sensitivity in clinical *P. aeruginosa* isolates remains equivocal. However, cross-resistance within antibiotic classes is common. Colistin therapy is promising since resistance to it was rare despite its intensive use in the studied patients.

## Teaser

We used Pseudomonas isolates from cystic fibrosis patients to assess associations between phenotypic resistance and sensitivity to other antibiotics, a minimal prerequisite for collateral sensitivity. Single patients contained multiple P. aeruginosa genotypes, which usually varied in resistance phenotypes. We did not find evidence for consistent phenotypic sensitivity of resistant isolates to other antibiotics.

## INTRODUCTION

Chronic bacterial infections typically require long-term antibiotic therapy. Sustained antibiotic deployment, however, drives the evolution of resistance, which progressively complicates treatment. This situation is exacerbated by the rapid spread of resistance genes [1], especially in Gram-negative bacteria [2], which makes new infections increasingly difficult to treat. There is thus a pressing need for treatment strategies that deploy antibiotics rationally and sustainably [3]. A possible answer to failing antibiotic regimes is the exploitation of collateral sensitivities of resistant bacteria [4–7], an approach which has shown great promise *in vitro*. The rationale is that bacteria that evolved resistance to one antibiotic can suffer evolutionary tradeoffs that cause increased susceptibilities to one or several other antibiotics. It may thus be possible to respond to resistance-induced treatment failure with carefully chosen antibiotics to which the resistant pathogen has now become more susceptible, potentially without extensive diagnostic screens.

The demand for sustainable antibiotic treatments is particularly pressing for cystic fibrosis (CF) patients, whose lives crucially depend on effective antibiotics. CF is an autosomal-recessive disease caused by a loss-of-function mutation in the cystic fibrosis transmembrane conductance regulator (CFTR) gene. Dysfunction of the CFTR channel in lung epithelia results in the secretion of viscous mucus that diminishes the ability of epithelial cilia to clear bacteria [8]. CF patients therefore suffer chronic pulmonary infections with a range of bacteria including *Pseudomonas aeruginosa*, *Staphylococcus aureus, Burkholderia cepacia* complex, *Haemophilus influenzae* and many other, often unculturable species [9]. As infections progress, *P. aeruginosa* gradually become dominant in the bacterial community and respond to increasingly aggressive antibiotic therapy with *de novo* evolution of drug resistance and increased abilities to form biofilms. In over 80% of patients, treatment eventually fails and lung damage caused by chronic infection and inflammation ends with lethal respiratory failure [10].

*Pseudomonas* causing chronic infections in CF have adapted to the complex and long-term evolutionary pressures of the host environment with antibiotics as one of the major selective forces [11]. Whether the collateral sensitivity concept applies to bacteria that have naturally evolved resistance remains unclear because previous studies focused on bacteria that were experimentally evolved to high-resistance levels *in vitro* [4, 7]. Testing the clinical applicability of a concept that was conceived under simplified laboratory conditions with a single selective constraint is necessarily challenging, because each clinical CF isolate has a unique evolutionary history involving diverse selective pressures, including multiple long-term exposures to various antibiotics. Nevertheless, a minimal prerequisite for candidate collateral sensitivities of phenotypically resistant genotypes would be reduced susceptibility to at least one other antibiotic. Clinically applicable collateral sensitivity thus requires the existence of consistent phenotypic sensitivities to antibiotics.

In this study we therefore investigated antibiotic sensitivity and resistance profiles covering 13 antibiotics of 4 classes of *P. aeruginosa* isolated from the lungs of CF patients using two clinical diagnostic tools (E-test and VITEK), and the broth dilution approach typically used in experimental evolution. Moreover, we used whole-genome sequencing to investigate which mutations may explain antibiotic resistance. We also used an MLST approach to assess within-patient clonal diversity and to infer whether isolates with similar resistance profiles were closely related. Finally, we related genetic and genomic changes to the resistance/sensitivity profiles and other patient data.

## METHODOLOGY

### Isolates and phenotypic characterization of *P. aeruginosa* clones from sputum samples

Clinical isolates were received from the Satteldüne rehabilitation clinic on the island of Amrum, Northern Germany between August and October 2014. Sputum samples were collected on the day of arrival (before the start of antibiotic treatment), after 1 week of antibiotic treatment in the clinic and on the day of departure after 4 weeks of treatment. We focused on 45 isolates from 13 patients who were chronically colonized with *P. aeruginosa* and were regularly treated with the antibiotics colistin or tobramycin. For five patients, one of these antibiotics was combined with ciprofloxacin for the duration of the stay (Table 1).

**Table 1.** Anonymized information on cystic fibrosis patients from whom *P. aeruginosa* were isolated for this study in the Sattelduene clinic on Amrum, Germany

Patient ID	Cohort	Age	Sex	Years since first PA diagnosis	Regular antibiotic treatment	Additional antibiotic treatment	Previous treatments
1	1	26	M	21	COL	CIP	IMI TOB CEF
2	1	38	M	10	TOB	–	TOB + MER
6	1	–	–	–	COL	CIP	AZT AZI
8	1	26	M	15	COL	–	CEF + TOB
10	1	41	M	6	COL	–	CIP
13	1	30	F	21	TOB	–	COL COL + CEF
201	2	28	F	15	COL	–	TOB + TAZ
203	2	20	F	11	COL	–	TOB + PIP
204	2	29	M	29	TOB/ − (cycle)	CIP	
205	2	38	M	28	COL	CIP	CEF + TOB
206	2	32	F	27	COL/TOB	CIP	TOB + TAZ CEF + TOB
207	2	22	F	8	COL/TOB	–	PIP + TAZ + TOB
210	2	29	F	–	TOB	–	CEF + TOB

The resistance phenotype (resistant, intermediate or sensitive compared with EUCAST reference concentrations) for each isolate was tested with the VITEK® system, which uses mass spectrometry to define resistance profiles.

### Dose response curves and MIC determination

To describe bacterial growth inhibition during antibiotic exposure, we obtained dose–response curves (DRCs) for 13 antibiotics of 4 antibiotic classes (Table 2). Bacteria were grown at 37°C in 96-well plates containing 10 different antibiotic concentrations in 8 replicates, 8 no-drug growth controls and 8 contamination controls (only medium) in a fully randomized design. After 12 h, we measured OD using a Tecan plate reader. For each drug and concentration, relative growth inhibition was calculated by comparing mean ODs of the eight replicates against the mean of no-drug control wells. Using the ‘drc’ package for R, we calculated a model curve describing the relationship between bacterial inhibition and antibiotic concentrations and extracted inhibitory concentrations (ICs), which express the degree of bacterial growth inhibition caused by specific drug concentrations in comparison with uninhibited growth.

**Table 2.** Overview of antibiotics and abbreviations used in this study

Name	Abbreviation	Antibiotic class	Stock solution
Aztreonam	AZT	Monobactams	50 mg/ml in DMF
Carbenicillin	CAR	Penicillins	50 mg/ml in 50:50 ethanol and H_2_O
Cefsulodin	CES	Cephalosporins (third generation)	20 mg/ml in H_2_O
Ceftazidime	CEF	Cephalosporins (third generation)	50 mg/ml in 0.1 M NaOH
Ciprofloxacin	CIP	Fluoroquinolones	25 mg/ml in 0.1M HCL
Colistin	COL	Polypeptides	50 mg/ml in H_2_O
Doripenem	DOR	Carbapenems	25 mg/ml in H_2_O
Gentamicin	GEN	Aminoglycosides	50 mg/ml in H_2_O
Imipenem	IMI	Carbapenems	5 mg/ml in H_2_O
Meropenem	MER	Carbapenems	20 mg/ml in DMSO
Piperacillin	PIP	Penicillins	50 mg/ml in H_2_O
Streptomycin	STR	Aminoglycosides	10 mg/ml in H_2_O
Tazobactam	TAZ	β-Lactamase inhibitor	10 mg/ml in H_2_O
Tobramycin	TOB	Aminoglycosides	50 mg/ml in H_2_O

### Broth-based resistance assays

To standardize comparisons of drug sensitivities, we established a reference framework consisting of ten antibiotic concentrations ranging between 1/16th of the PA14 reference minimum inhibitory concentration (MIC) to 32-times the PA14 MIC (Supplementary data, Table S1). Each clinical isolate was challenged against these 10 reference concentrations for each of 10 antibiotics (CAR, PIP, IMI, DOR, CEF, CIP, COL, GEN, STR, TOB). Exposure to aztreonam provoked filamentous growth that did not allow reliable DRC calculation; therefore AZT was excluded from the broth assay. In total, 4 replicates of 55 samples (46 clinical isolates, 7 evolved PA14 mutants and two PA14 controls) were challenged against 10 antibiotics on 325 96-well plates. DRC were inferred as described above.

### E-tests

Clinical bacterial strains are classified as ‘sensitive’, ‘intermediate’ or ‘resistant’ by comparing empirically obtained MIC concentrations to clinical cutoff values, which are maintained and revised by the European Committee on Antimicrobial Susceptibility Testing (EUCAST [12]). The Etest® strips (bioMérieux) were applied to MH agar plates inoculated with bacterial suspensions and incubated at 37°C for 24 h. The experiment was performed in triplicate for each strain/antibiotic combination. The MIC was determined as the next highest scale value above complete growth inhibition.

### Experimental evolution of colistin resistant mutants

Two separate 96-well-plates containing 20 replicates of IC_50_ concentrations of colistin, no-drug and empty controls in a randomized setting were incubated at 37°C under constant shaking. After 12 h of incubation, 50% of the population from every well was transferred to a freshly prepared plate with the same design. The concentration of each antibiotic was increased by 25–50% of the previous concentration at every fourth transfer, depending on the OD measurements of the previous transfer. A mild increase was chosen for very low ODs to avoid extinction. A strong increase was chosen for high ODs to strengthen the selective regime. The experiment was carried out for a total of 58 transfers.

### DNA extraction and whole genome sequencing

DNA was extracted using the DNeasy Blood and Tissue preparation kit (QIAGEN) according to the manufacturer’s recommendations. The DNA extracts were sequenced by the sequencing facility at the Institute of Clinical Molecular Biology, Kiel University Hospital, using the Illumina HiSeq paired-end sequencing technology with an insert size of 350 bp at 300× coverage.

### Analysis and SNP detection

To correct sequencing errors, clip adapters and remove unreliable reads, the raw reads were first processed in Skewer [13]. We called single nucleotide polymorphisms directly from raw reads using discoSNP ++ [14]. The resulting SNPs were locally aligned to the PA14 reference genome and annotated using snpEff and the *Pseudomonas* genome database [15]. SNP statistics were further calculated in R.

### Functional term analysis

The collated gene list of all samples was analyzed using the gene clustering and functional term enrichment approaches in DAVID 6.7 (http://david.abcc.ncifcrf.gov/ 24 May 2016, date last accessed) [16] with *Pseudomonas aeruginosa*_UCBPP_PA14_uid57977 as background. As annotations we used a combination of GO terms, protein–protein interactions, protein functional domains, disease associations, bio-pathways (KEGG + PANTHER), sequence features, homology, gene functional summaries, gene tissue expression and manual annotations from the literature encoded in DAVID.

### Mapping of MLST and resistance genes

To estimate the phylogenetic relationships among the isolates, we mapped the reads of each sample to a set of 13 PA14 housekeeping genes [17, 18] that are used for MLST (multi-locus sequence typing) typing of pseudomonads. The genes included were *acsA*, *adhA*, *aroE*, *aph*, *bdhA*, *guaA*, *mtlD*, *mutL*, *nucP*, *nuoD*, *ppsA*, *trpE* and *xdhB*. Similarly, we mapped all reads to a set of genes that were known to be commonly involved in antibiotic resistance [19,20], consisting of the genes *ampC*, *ampD*, *emrB*, *gyrA*, *gyrB*, *ftsI*, *fusA*, *mpl*, *mexR-mexaAB-oprM*, *oprN-mexF-mexE-mexT*, *oprJ-mexD-mexC-nfxB-morA*, *oprD*, *pmrB*, *rhlRI*, *lasRI*, *parC*, *pyrE*, *pvdS*, *pvdG*, *pvdL*, *retS*, *pmrA*, *pmrB*, *rpoS*, *recA*.

For each sample, reads were mapped to the reference sequences using Burrows-Wheeler Aligner with a maximum edit distance of 1 in the seed, the fraction of missing alignments (given a 2% base error rate) set to 0.01 and subsequently aligned using the sample module. We called SNPs on the bam alignments using VarScan 2 [21].

### Phylogenetics

The resistance genes and MLST alignments were analyzed using RaxML 8 [22] and MrBayes 3.2 [23]. In RaxML, we did 500 maximum likelihood searches given a general time reversible model of molecular evolution with γ-distributed rates and disparate random initial seeds with 10 000 bootstraps. In MrBayes, we specified a general time reversible 4 × 4 nuclear model with 6 rate categories, rates drawn from a γ distribution and an estimated proportion of invariable sites and haploid genomes. We ran the analysis for 6 million generations with 25% burn-in, after which chain divergence dropped below 0.001 in all analyses.

## RESULTS

### Resistance and sensitivity profiles

**Figure 1.**
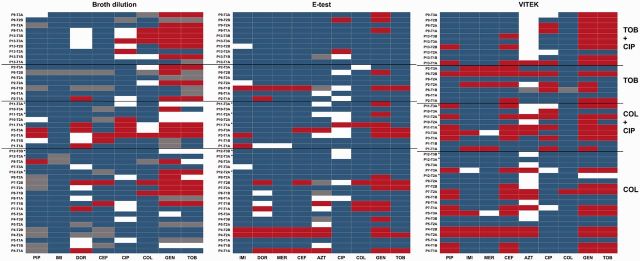
Resistance profiles of clinical isolates compared with EUCAST resistance breakpoints based on (**a**) broth dilution; (**b**) E-test and (**c**) VITEK. Heat maps represent categories resistant (red), intermediately resistant (white), sensitive (blue) or not measured (grey). Asterisks indicate COL-TOB cycling. Abbreviations on the left describe previous treatment of patients. See Table 1 for abbreviations of antibiotics

To obtain resistance profiles for 13 antibiotics for each of the 45 clinical isolates, we used three methods. First, we determined dose–response curves with the relative PA14 MIC reference concentrations for each antibiotic (see supplement). Second, we performed E-tests that measure MICs as the minimal concentration on the strip where a bacterial zone of inhibition can be seen. For both methods, we compared resistance levels with EUCAST cutoff values, and standardized the measured MIC data to the PA14 laboratory strain. Third, we also used the VITEK mass spectrometry system to directly measure EUCAST resistance breakpoints. For each type of data we constructed a heatmap depicting the relative resistance of each isolate against all drugs tested (Fig. 1 and Supplementary data, Fig. S1). Finally, we assessed all possible pairwise correlations among all antibiotic resistances using Pearson’s correlation coefficient.

### Broth dilution

Using the broth dilution method, most of the clinical isolates showed intermediate to high resistance compared with PA14, especially to the aminoglycosides streptomycin (77% of samples more resistant), gentamicin (73%), tobramycin (71%), to the penem doripenem (69%) and the fluoroquinolone ciprofloxacin (64%) (Supplementary data, Fig. S1a). Lower resistance compared with PA14 was observed against cefsulodin (22% resistant), piperacillin (13%) and imipenem (6%). Resistance against colistin was also low (37% resistance). All but one sample were sensitive to carbenicillin, but this result must be taken with caution, because our PA14 reference strain was itself intermediately resistant to carbenicillin according to EUCAST. Compared with EUCAST breakpoints, the most frequent resistance was to aminoglycosides (Fig. 1a).

**Figure 2.**
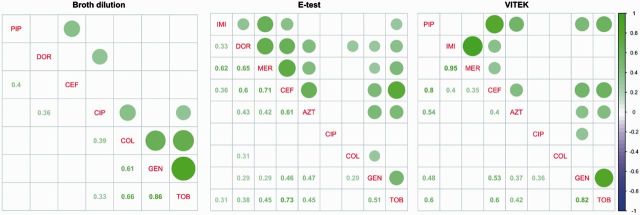
Pairwise correlations of resistances among antibiotics based on EUCAST resistance data using Pearson’s correlation coefficient *ρ*. Only statistically significant correlations (*P* < 0.05) and corresponding values of *ρ* are shown. Purple circles, sensitivity; green circles, resistance. Circle sizes graphically depict strengths of correlations

Based on our pairwise correlation analysis (Fig. 2a), we found significant cross-resistance among the three aminoglycosides gentamicin, tobramycin and streptomycin (GEN-TOB: 0.946; GEN-STR: 0.911; TOB-STR: 0.908; all *P* < 0.01). Additionally, we found evidence for cross-resistance between colistin and these aminoglycosides (*ρ* = 0.7; *P* < 0.01). However, we could not find any negative correlation between resistances to any two antibiotics in these samples.

### E-tests

We sometimes found surprising variation among the technical replicates performed for a particular sample using the E-test. In particular, six samples showed more than one scale unit variation among the three performed replicates. Another 15 cases showed a variation of one scale unit that would cross a EUCAST beakpoint-i.e. resulting in the same sample being either classified as resistant or sensitive.

The highest frequency of EUCAST-defined resistances was encountered for gentamicin (43.7% of tests, 46.7% of strains; Fig. 1b). Interestingly, resistance against colistin and tobramycin, the two drugs used regularly by all patients, was only found in 10% of the samples. Merely one patient carried a *P. aeruginosa* isolate that was highly resistant to colistin. The remaining antibiotics, ceftazidime and the three carbapenems, also had resistance rates well below 10%.

However, when we compared resistance levels relative to the PA14 lab strain (rather than to EUCAST values), we found that almost all clinical isolates were more resistant to imipenem (93%), doripenem (91%), meropenem (55%), ciprofloxacin (84%), gentamicin (82%) and tobramycin (91%) (Supplementary data, Fig. S1). The higher proportion of resistances in this comparison is not surprising because PA14 was chosen as a wild-type representative without clinical resistances (except intermediate resistance to aztreonam and carbenicillin). Nevertheless, 68% of samples were still more sensitive to ceftazidime and 80% more sensitive to aztreonam than PA14.

The pairwise correlation analysis of the E-test data (Fig. 2b) revealed cross-resistance among all β-lactam antibiotics (imipenem, doripenem, meropenem, ceftazidime, aztreonam; Spearman’s *ρ* between 0.50 and 0.86; *P* < 0.05) and among the aminoglycosides gentamicin and tobramycin (*ρ* = 0.8, *P* < 0.05). Again we did not identify any negative correlations among the measured resistances.

### VITEK

The VITEK scan directly classifies samples into the three EUCAST categories (Fig. 1c). The highest proportion of resistances was found to gentamicin (71%) and tobramycin (62%). Interestingly, all samples resistant to tobramycin were also resistant to gentamicin. Further resistance frequencies were moderate: 55% of the isolates were resistant to ceftazidime and 44% to piperacillin, but only 28% to aztreonam, 28% to ciprofloxacin, 17% to imipenem and 13% to meropenem. Finally, only one sample showed resistance to colistin.

The correlation matrix for this method showed strong positive correlations for meropenem and imipenem (*ρ* = 0.95, *P* ≤ 0.01) and for gentamicin and tobramycin (*ρ* = 0.82, *P* < 0.01; Fig. 2c). Again, we did not recover any strong negative correlations that would suggest consistent sensitivity of any of these clinically resistant isolates to another antibiotic.

### Correlations with patient data

To determine whether the resistance profiles could be explained by any of the patient factors, we constructed a generalized linear model for each antibiotic with resistance as a response variable, cohort, week, primary treatment (COL or TOB) and secondary treatment (addition of CIP) as fixed variables and patient number and patient age as random variables. Our model search identified a statistically significant influence of tobramycin treatment on tobramycin resistance, but not on any other resistance. For colistin, none of the treatments significantly influenced resistance (Supplementary data, Fig. S5). These analyses confirm the lack of consistent negative correlations among resistances to sets of antibiotics in these clinical isolates, despite their long-term exposure to antibiotic treatment with tobramycin and colistin, and despite the fact that 20 of them (44%) have evolved clinical resistance to at least one antibiotic and 11 were multi-drug resistant.

### Genomic analyses

**Figure 3.**
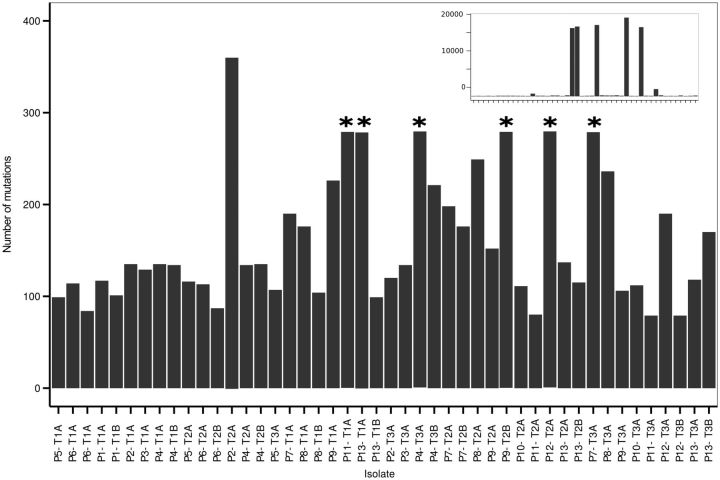
Number of SNPs and indels for clinical isolates compared with PA14. Stars indicate truncated bars. Strains with high numbers of differences to PA14 are shown in the inlay (note increased range of *Y* axis)

To investigate the genetic adaptations and inter-relationships of the clinical isolates, we sequenced whole genomes of all clinical isolates using Illumina paired-end sequencing. The SNP and indel analysis revealed an average of 188 mutations per strain (excluding the six strains for which many mutations were found), many of which were frame-shift mutations (Fig. 3). We identified six divergent strains which differed at 19 208, 17 387, 17 020, 16 869, 16 640 and 1745 positions from the PA14 reference, respectively. These could be mutator strains because they all carry non-synonymous (in three samples frame shift) mutations in two or more of the mismatch repair genes *mutL*, *mutS*, *mutT* or *mutM* genes, which are known to be involved in hypermutation [24]. Nevertheless, it cannot be excluded that the high numbers of mutations result from mapping to a distantly related reference strain. For the other isolates, the genes that received most non-synonymous mutations across the samples were related to Type VI secretion, ABC-type membrane transporters, glutamine synthetases and the BaeS two-component system involved in drug resistance. Most samples carried at least one mutation that could be related to antibiotic resistance. For annotations of all mutations found in this study (see Supplementary data, Table S2).

Because non-synonymous and frame shift mutations are most likely to change the function of proteins, we subjected the list of all genes with at least one such mutation in at least one isolate to functional annotation and gene clustering analysis in DAVID. We found significant enrichment of mutations in transcription factors (enrichment score 6.6, *P* < 10 ^−^^8^), cell wall synthesis and alginate production (ES 3.01, *P* < 10 ^−^^6^), two-component sensors (ES 1.79, *P* < 10 ^−^^5^) and porin complexes (ES 2.73, *P* < 0.01). We also clustered genes according to their functional classification. This yielded several significant clusters: two component sensors, alginate (ES 3.58) and phenazine production (ES 2.44), multi-drug resistance (ES 0.26) and 30S/50S ribosomal genes (ES 1.58). A more comprehensive study of *Pseudomonas* adaptation to the CF lung identified antibiotic resistance; motility/attachment; DNA replication, recombination, modification and repair; cell wall/LPS/capsule; secreted factors; and transcription factors as key functions [19]. All of these categories were enriched in our data, although not all significantly, potentially due to the much smaller sample size in our data. That same study identified a set of pathoadaptive genes, ∼50% of which was also mutated in our data (e.g. *mex* and *pil* operons, *vgrG*, *pvdG*, *algU*, *gyrA*, *gyrB*, *mpl* and *oprD* genes). Overall, these results suggest that the strains may have adapted to the lung environment, and also carry a substantial number of resistance mutations that likely reflects their long-term exposure to antibiotics.

### Phylogenetic relationships

**Figure 4.**
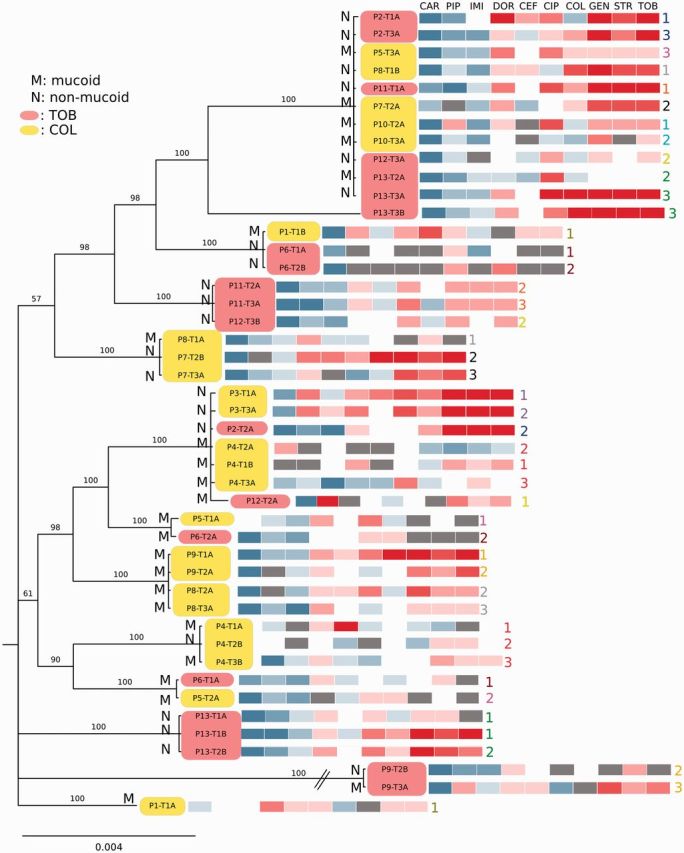
Phylogenetic interrelationships among clinical isolates based on 13 MLST genes. Bayesian phylogram based on a GTR + Γ + I model of molecular evolution, 6 million generations of MCMCMC with 25% burn-in. Nodes are labeled with posterior probabilities. The phylogeny is annotated with the EUCAST E-test resistance profiles as in Fig. 1b. Yellow and magenta boxes indicate isolates from patients treated with colistin and tobramycin, respectively. M, mucoid; N, non-mucoid. Numbers in corresponding colours express order in which isolates were taken from the same patient at consecutive time points

The Maximum Likelihood and Bayesian trees were similar, with high bootstrap and posterior probability support for most nodes, except for the most basal ones (Fig. 4 and Supplementary data, Fig. S3). The six isolates containing high numbers of mutations did not cluster together, but were distributed across the tree. This likely represents the phylogenetic position of their core genomes, suggesting they evolved independently from one another. The phylogenies identified 12 clonal lineages, of which 3 were unique to 1 patient and 6 only occurred in 2 patients. One widespread clone was found in 8 of the 13 patients. The trees further suggest that most patients carried multiple genetically distinct clones. Moreover, four patients had one clone that was consistently sampled across multiple time points; another even had two consistently reoccurring clones. These confirm the chronic nature of *Pseudomonas* infection and the limited success of eradication with antibiotic treatment. Nine patients showed high clonal variation across time points, but with only limited overlap between consecutive time points. We could not evaluate whether this variation was due to a lack of sampling depth or to clonal turnover within the lung.

Mapping of phenotypic data onto the MLST phylogeny revealed that (i) the most resistant profiles did not cluster together; (ii) identical clones recovered from different patients did not necessarily have similar resistance phenotypes; (iii) resistance profiles of clones recovered at different time points from single patients did not change profoundly and (iv) within single patients at least one mucoid, sensitive and one non-mucoid, more resistant clone coexisted. Taken together, this shows that antibiotic resistance is mostly determined by specific genes, which is not reflected in the evolutionary history of individual lineages. We therefore constructed phylogenies using a set of genes known to be involved in antibiotic resistance (the resistome). Although this tree was dissimilar from the MLST tree (Supplementary data, Fig. S4), it did not group the most resistant samples. We conclude that resistance profiles were most likely determined by individual gene combinations.

### Experimental evolution of colistin resistance

Although half of the patients received colistin treatment, we could only find one colistin-resistant clone. We therefore tested whether PA14 can experimentally evolve resistance to colistin. The experiment yielded seven clinically resistant colistin strains with MICs above 32× of the PA14 ancestral MIC. Compared with the ancestral PA14 strain, all of the evolved lineages showed at least 4× increased MICs to aminoglycosides and at least 2× increased MICs to doripenem (Supplementary data, Fig. S2). Interestingly, we additionally observed collateral sensitivity to carbenicillin, piperacillin, imipenem and ciprofloxacin in all of these lines (Supplementary data, Fig. S2).

## DISCUSSION

This study aimed to determine whether phenotypically resistant clinical pathogen isolates exhibit consistent sensitivities to other antibiotics. This type of evidence is a minimal requisite for the application of the collateral sensitivity concept to clinical isolates. This idea comes from *in vitro* evolution experiments with *E. coli*, which showed that a majority of bacterial lineages that evolved high resistance to one antibiotic concomitantly showed increased susceptibility to at least one other antibiotic [4, 7]. Subsequent exposure to a drug to which the resistant bacteria have been sensitized would thus eradicate resistant mutants more effectively than subpopulations that did not become resistant. Therefore, information on collateral sensitivities may theoretically be exploited in the clinic to devise therapies that counter resistance.

To address this important issue, we turned to clinical isolates of cystic fibrosis patients that have adapted to long-term antibiotic therapy. Although the concept of collateral sensitivity has been formulated based on phenotypic results, it is usually assumed to be based on pleiotropic effects of the resistance-causing gene. For clinical isolates, we cannot directly test the involvement of specific resistance genes with such pleiotropies. Nevertheless, we are able to assess whether single clinically resistant genotypes showed correlated patterns of phenotypic resistance and sensitivity among different drugs. We found that 90% of isolates were (phenotypically) intermediately resistant to at least one antibiotic, of which 40% were clinically and 15% multi-drug resistant. This was supported by our genomic analysis; all of our isolates possessed non-synonymous mutations in at least one gene known to be involved in antibiotic resistance (for example, *oprD*, *gyrA* or *B*, *mex*-type efflux systems, *parC*, *rpoB*, *baeS*). Moreover, the GO analysis highlighted (among others) genetic changes related to resistance in genes encoding or regulating expression of porins, outer membrane permeability, multi-drug efflux pumps, gyrases and drug modifying enzymes (aminoglycoside modification enzymes and/or β-lactamases). Therefore, our material represented a range of clinically problematic resistance levels for which the discovery of collateral sensitivity would be most relevant. However, none of three methods of determining resistance uncovered significant correlations between resistances to one antibiotic and consistent sensitivities to another.

The discrepancy between our results and those obtained from *in vitro* experiments is striking. For example, *E. coli* that evolved resistance to gentamicin *in vitro* are collaterally sensitive to several other antibiotics, including colistin and cefuroxime (a β-lactam similar to cefsulodin in our study) [4]. This was plausibly explained by the antagonistic effects of aminoglycoside-resistance mutations [7]. These cause a reduced membrane potential, which in turn lowers active uptake of aminoglycosides and alleviates drug-induced oxidative stress. But they also diminish the activity of other efflux pumps, which renders the resistant strains more susceptible to antibiotics of other classes that accumulate in the cell. In our clinical data set, however, gentamicin-resistant samples either showed no correlations or consistent cross-resistance to colistin and β-lactam antibiotics (including cefsulodin). Similarly, when we experimentally adapted *P. aeruginosa* to colistin under controlled laboratory settings, it did express collateral sensitivities to other antibiotics. Yet colistin-resistant clinical strains did not show consistent sensitivities to other drugs.

These observations may suggest that costly mutations causing evolutionary tradeoffs *in vitro* simply do not occur *in vivo*, because natural evolution of CF strains involves adaptations to additional selective pressures which preclude the type of mutations seen in artificially selected lines. Alternatively, collateral sensitivity is a transient phenomenon that can be observed in laboratory experiments with strains naive to antibiotics, but that may disappear during chronic exposure to drugs because compensatory evolution or adaptation to different antibiotic exposures has already lifted their effects.

Moreover, compared with *E. coli*, *Pseudomonas* has a large genome that is highly adaptable to multiple environments. It possesses multiple intrinsic resistance mechanisms such as an inducible AmpC β-lactamase, a wide range of efflux pumps and reduced membrane permeability. Therefore, *Pseudomonas* is naturally resistant to a wide range of antibiotics [20]. The type of evolutionary tradeoffs that cause collateral sensitivity between aminoglycosides and other classes of antibiotics in *E. coli* may thus not exist in *P. aeruginosa*. A last possibility is that these tradeoffs could not be uncovered with the limited sampling, the lack of power to directly detect pleiotropic effects for particular gene mutations or the choice of antibiotics in our study, although these antibiotics represented the four antibiotic classes that are clinically used for treating *P. aeruginosa* infections (and that showed collateral effects in other studies). Taken together, further studies are needed to determine whether collateral sensitivities can be found in other types of *Pseudomonas* infections, or for other clinical isolates of multi-drug resistant Gram-negative species such as *Klebsiella* sp. or *E. coli*. Ideally, such studies should focus on clonal lineages that were exposed to few antibiotics for a longer time, with demonstrable genetic adaptation to those antibiotics and minimal confounding adaptations to other evolutionary pressures.

In this study, we did uncover strong positive resistance correlations among antibiotics of particular classes, for example those belonging to the aminoglycosides (tobramycin and gentamicin), β-lactam antibiotics (imipenem, doripenem and cefsulodin; cefsulodin or ceftazidime and aztreonam), and between aztreonam (a monobactam) and the aminoglycosides. Multi-drug resistances could be inferred from combinations of mutations: (i) resulting in overexpression of several multi-drug efflux pumps; (ii) in outer membrane porins, β-lactam acylases and in enzymes and structural components involved in peptidoglycan stability (targets of β-lactams); (iii) gyrase mutations (targets of fluoroquinolones); and (iv) aminoglycoside phosphotransferases and –acetylases. Because isolates contained many mutations, we cannot rule out that resistance to the different drugs results from independent mutations, rather than collateral resistance. The availability of a demonstrable plethora of resistance mechanisms in the *Pseudomonas* genome may increase the number of evolutionary routes this bacterium may take to multi-drug resistance, and may increase the likelihood of multi-drug resistance in this pathogen.

We further found that all but one of the isolates were sensitive to colistin despite its heavy therapeutic use in half of these patients. Colistin, a member of the polymyxin class, has been abandoned for general treatment of Gram-negative infections in the 70s on grounds of high neuro- and nephrotoxicity, although this view is increasingly being challenged [25]. In CF, colistin has been commonly used for the lack of alternatives, and recently it has been through a revival as a last resort therapy for multi-drug resistant Gram-negative infections. Interestingly, resistance to colistin seems to be much rarer than to other antibiotics in the hospital environment. However, this is not a result of the intrinsic inability of *Pseudomonas* to evolve resistance, since our evolution experiment showed that high levels of resistance are easily reached *in vitro*. Moreover, our experimentally evolved lines were collaterally sensitive to carbenicillin, piperacillin, imipenem and ciprofloxacin. Why the incidence of colistin resistance in clinical isolates is rather low remains a matter of speculation. It is possible that colistin-resistant *P. aeruginosa* cannot evolve or survive in the CF environment. However, we caution that generally low colistin resistance may be a side-effect of limited use of polymyxins over the last decades. Therefore, colistin should be treated as a cherished compound reserved for specific cases. Otherwise, it may turn out to be another magical bullet that eventually fails in the face of bacterial resistance evolution.

Our phylogenetic analyses further highlight that many patients harbor multiple *P. aeruginosa* clones with different phenotypic properties, and that these properties did not change in response to antibiotic treatment. Moreover, different isolates of the same clone showed distinct phenotypic properties, whereas unrelated genotypes could display similar phenotypes. Evidence for complex phenotypic variability that seems robust to antibiotic therapy now abound in studies where sputum samples were more deeply sampled [26–29]. This implies that current clinical sampling practices, which typically test two or three morphologically distinct *P. aeruginosa* clones, underestimate the biological diversity within a patient’s lung community, potentially resulting in biased antibiograms and misguided treatment decisions. It is thus highly recommended that multiple *P. aeruginosa* clones be included in susceptibility testing. One study even demonstrated that mixing randomly picked colonies before susceptibility testing leads to consistently higher resistance levels than obtained for single colonies [30]. To avoid the paradoxical situation in which antibiotic treatment favors resistant clones by removing competition from susceptible strains, we strongly urge for a broader sampling regime that more readily captures the resistance properties of the bacterial community. A promising avenue is next generation sequencing, which offers the advantage of covering the full bacterial diversity of clinical samples, including unculturable or rare species. However, it is expensive when compared with culture-based methods, requires considerable expertise, and the reliability and repeatability of the method has been insufficiently tested. Moreover, it requires full understanding of the gene repertoire that can contribute to resistance, in order to allow reliable antibiotic resistance profiling. Nevertheless, we are confident that new technologies allowing comprehensive sampling of bacterial communities and determination of phenotypic properties from entire communities will benefit cystic fibrosis diagnostics in the future.

## CONCLUSION

*Pseudomonas aeruginosa* that naturally evolved antibiotic resistance during chronic lung infections in CF patients exhibited high cross-resistance within antibiotic classes and between aminoglycosides and monobactams. Negative correlations indicating resistance to one antibiotic and sensitivity to a second antibiotic could not be identified, in contrast to *in vitro* studies. The low incidence of colistin resistance despite its use in the studied patients deserves further scrutiny, especially since colistin resistance easily evolved under laboratory conditions. Clinical sampling regimes based on few clones may underestimate *P. aeruginosa* clonal diversity within the CF lung. Moreover, we found that multiple genetic lineages may have similar resistance profiles, whereas closely related clones may differ in their resistances.

## SUPPLEMENTARY DATA

Supplementary data is available at *EMPH* online. 

## Supplementary Material

Supplementary Data
