# Supplementary figures and images for: Association between clinical antibiotic resistance and susceptibility of Pseudomonas in the cystic fibrosis lung
Source: Evol Med Public Health. 2016 May 21;2016(1):182–94. doi: 10.1093/emph/eow016 (PMC4906436; doi:10.1093/emph/eow016)

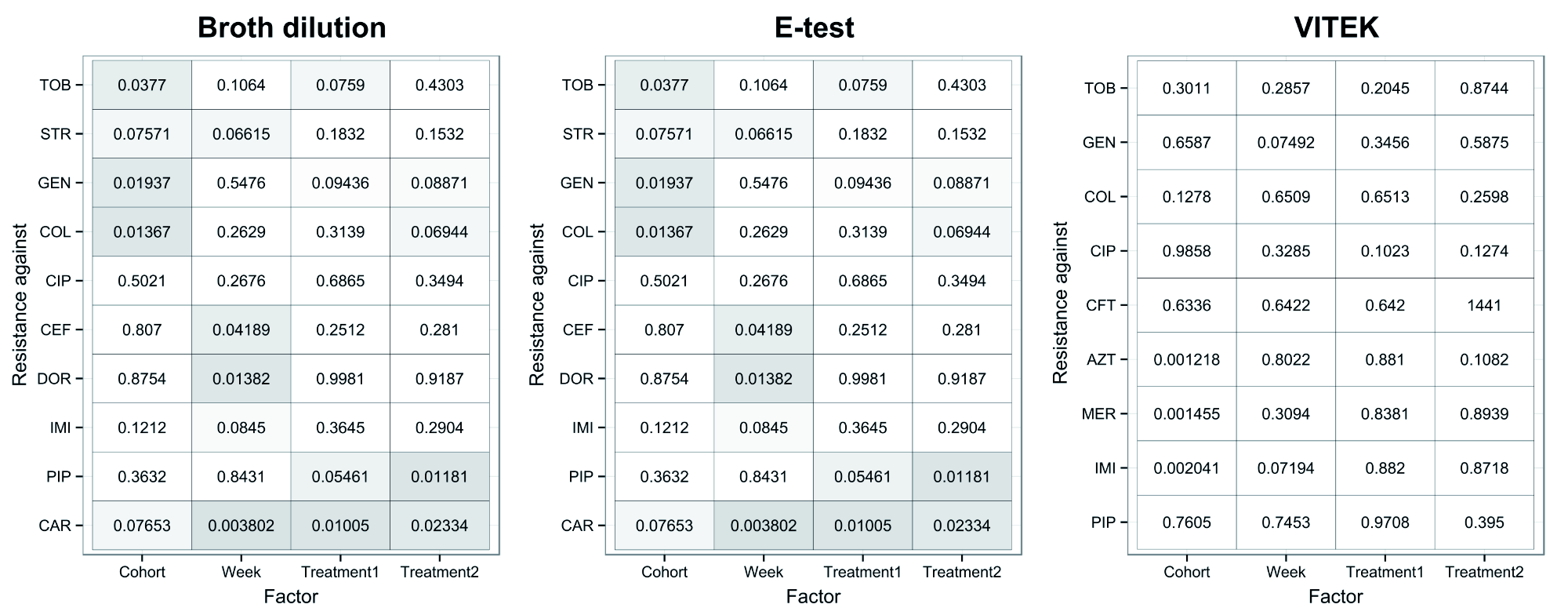

Supplement: Supplementary Data [file eow016_Supp.zip › figS5.tif]

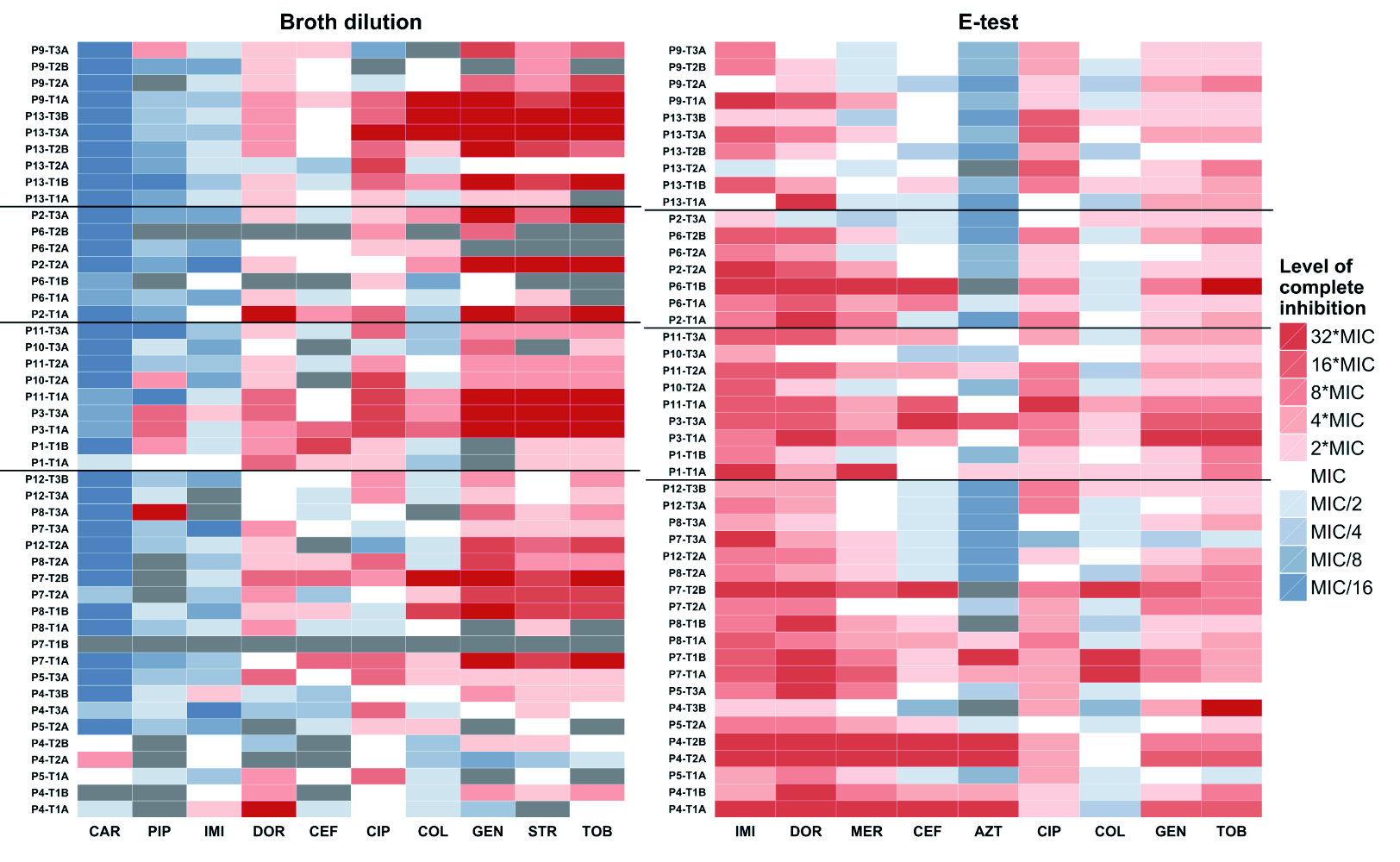

Supplement: Supplementary Data [file eow016_Supp.zip › figS1.tif]

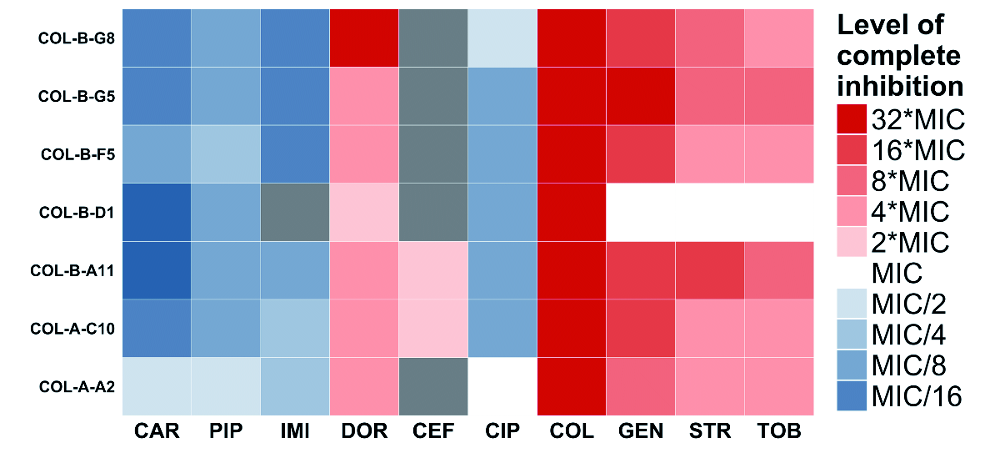

Supplement: Supplementary Data [file eow016_Supp.zip › figS2.tif]

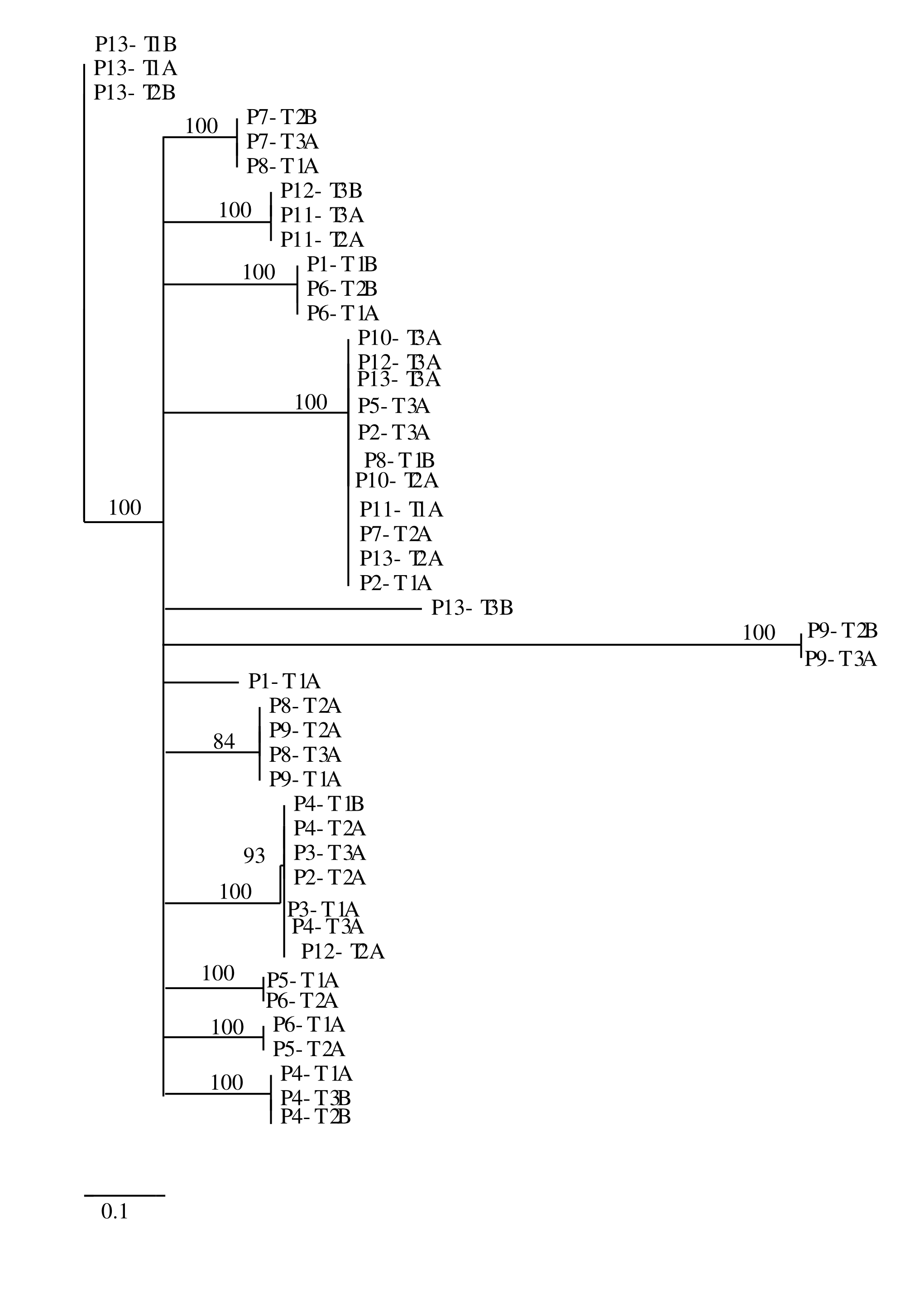

Supplement: Supplementary Data [file eow016_Supp.zip › figS3.tif]

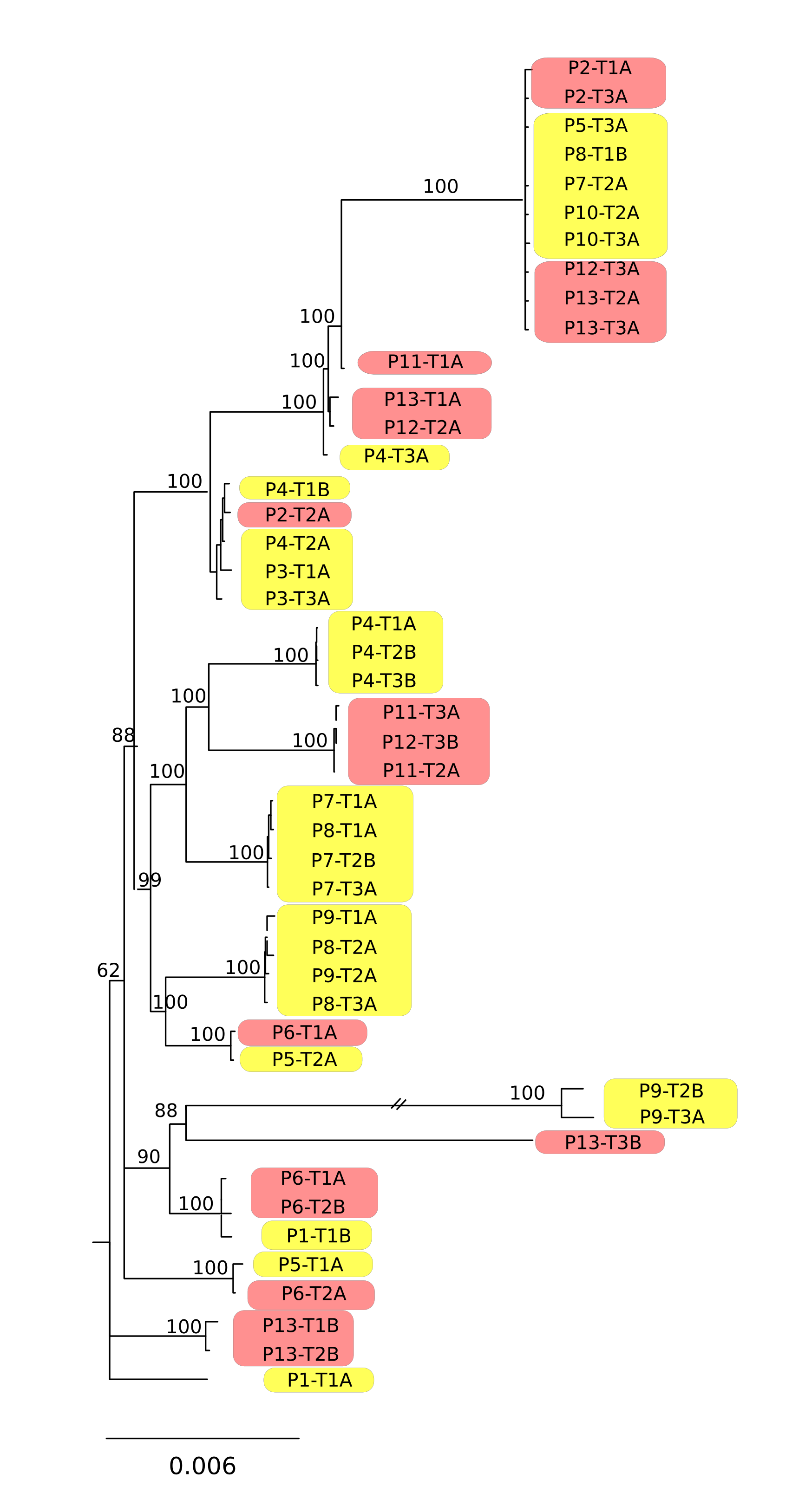

Supplement: Supplementary Data [file eow016_Supp.zip › figS4.tif]
